# Association of body composition indicators with colorectal cancer: a hospital-based case-control study

**DOI:** 10.1007/s00432-024-05866-4

**Published:** 2024-07-09

**Authors:** Qiujin Chen, Kai Li, Yang Liu, Xiaozhai Yu, Fengrong Ou

**Affiliations:** 1 Department of Clinical Nutrition, The First Hospital of China Medical University, Shenyang, 110001 China; 2 Department of Immunization, The Affiliated Wuxi Center for Disease Control and Prevention of Nanjing Medical University, Wuxi Center for Disease Control and Prevention, Wuxi, 214023 China; 3 Department of Surgical Oncology and General Surgery, The First Hospital of China Medical University, Shenyang, 110001 China

**Keywords:** Colorectal cancer, Body composition, Obesity, Cachexia, Malnutrition

## Abstract

**Purpose:**

Colorectal cancer (CRC) is a common malignancy that affects adults worldwide, causing a high disease burden. Few studies have examined the relationship between body composition (BC) measures and the prevalence of CRC. Our purpose was to investigate the relationship between pertinent BC indicators and CRC.

**Methods:**

Bioelectrical impedance analysis, laboratory test results, face-to-face questionnaire investigation, and nutritional risk assessment (Nutritional Risk Screening 2002 and Patient-Generated Subjective Global Assessment) were used in this case-control study. Bioelectrical impedance analysis in the case group was performed prior to antitumor therapy/surgery.

**Results:**

From June 2018 to January 2019, a total of 303 cases and 286 controls were included. The results showed that low body fat percentage (BFP) and high visceral adiposity index (VAI) groups had a higher risk of developing CRC in comparison to the normal BFP and normal VAI groups. The risk of CRC decreased with the increase of BFP. The group with a normal BC had a lower risk of developing CRC compared to those with a greater VAI and a lower BFP, as indicated by the results of the pairwise and total combinations of VAI, fat-free mass index (FFMI), and BFP. Additionally, FFMI and VAI had positive correlations with prealbumin, serum albumin, and nutritional risk scores.

**Conclusion:**

Low BFP and high VAI are associated with higher CRC risk. FFMI and VAI are positively correlated with prealbumin, serum albumin, and nutritional risk scores in CRC patients.

**Supplementary Information:**

The online version contains supplementary material available at 10.1007/s00432-024-05866-4.

## Introduction

Colorectal cancer (CRC) is a common malignant tumor that includes colon cancer and rectal cancer. The incidence and mortality of CRC are among the top common malignant tumors (Sedlak et al. 2023; Wang et al. 2023). In 2020, the World Health Organization (WHO) conducted a survey on the incidence and mortality of 36 different types of cancer in 185 countries. The results revealed that there were over 1.93 million new cases of CRC and over 935,000 deaths globally, placing the disease third and second in terms of global incidence and cause of death, respectively (Sung et al. 2021). Furthermore, new research revealed that the incidence and mortality of CRC have been rising, with an estimated 3.2 million new cases and 1.6 million deaths from the disease by 2040 (Morgan et al. 2023; Patel et al. 2022; Xi et al. 2021; Zygulska et al. 2022). Combining these results, we may conclude that CRC risk will become increasingly severe, and the resulting disease burden will also become more severe. As a result, inhibiting the genesis of CRC is crucial for preventing its creation. It is crucial to look into the related causes of CRC, determine the main risk factors for CRC, and use this knowledge as a springboard for CRC treatment and prevention.

With the improvement of people’s living standards, obesity has become one of the serious public health problems. Body mass index (BMI) is a commonly used index to assess obesity, and BMI ≥ 30 kg/m^2^ is considered obese. However, BMI does not specifically distinguish between fat tissue and muscle mass, nor does it distinguish between fat accumulation in different body parts (Rontogiann et al. 2024). Obesity is an abnormal or excessive accumulation of body fat that may damage health (Oliveira et al. 2023). It is also a kind of physiological dysfunction caused by environmental, genetic, and endocrine factors (Mármol et al. 2017). Research showed that obesity and overweight put an additional strain on internal organs and increased the risk of several diseases, including cancer, chronic obstructive pulmonary disease, cardiovascular disease, and type 2 diabetes mellitus (T2DM) (Conway and Rene 2004). The occurrence and development of CRC are also multi-factor and complicated process steps and stages, which are affected by genetic factors and environmental factors (Vernia et al. 2021). The relationship between obesity and CRC has gained attention in recent years due to the growing body of research on the topic. The World Cancer Research Fund and the American Institute for Cancer Research have both identified obesity as a potential cause of CRC (Clinton et al. 2020; Lauby-Secretan et al. 2016). Measures characterized by abdominal obesity (Waist Circumference /Waist-to-hip ratio) are associated with the development of colorectal cancer, especially in men (Rontogiann et al. 2024). This shows that body part obesity has begun to be paid attention to, but waist circumference and waist-to-hip ratio are still measurements of the body surface, and can’t reflect the true accumulation of body fat content.

BC (body composition) refers to the content and distribution of different body components in the total body mass, including the content and distribution proportion of fat, muscle, bone, inorganic salts, and water in the human body, that is, the proportion of body fat tissue and non-fat tissue in the body weight. Bioelectrical impedance analysis is the most widely used and quick technique for determining BC without causing harm to the human body (Deutz et al. 2019). The typical BC analysis indicators, BFP (body fat percentage), VAI (visceral adiposity index), and FFMI (fat-free mass index), have been used extensively in the research of obesity and disease in recent years. BFP is the weight of body fat relative to body weight. The fat that surrounds internal organs is referred to as visceral fat. The VAI is computed using visceral fat area, which is expressed as visceral fat area (cm)/10. Nonfat weight without body fat is known as fat-free mass, and FFMI is computed by dividing fat-free mass by the square of height. The distribution of body fat is an important determinant of health. Multiple studies have shown that there is a relationship between BC and the occurrence of various diseases, including cancer, obesity, diabetes, aging, cardiovascular injury, et al. (Henriksson et al. 2022; Liu et al. 2021; Yuan et al. 2023).

In the past, overweight and obesity were mainly determined by BMI. Most studies have shown that obese BMI is related to the occurrence of CRC (O’Sullivan et al. 2022). In recent years, studies have shown that high lean body mass could promote health, and high BMI could reduce the risk of death, which is contradictory to the previous conclusion that high BMI could lead to diseases, causing the “obesity paradox”. Consequently, there has been doubt over the validity of BMI in identifying overweight and obesity (Bray 2023; Martin et al. 2013). Therefore, a more comprehensive approach is necessary to identify and evaluate overweight and obesity and to determine the risk of associated diseases. There is variation in the corresponding BC amongst persons with the same BMI. Furthermore, research has demonstrated that, for a given BMI, the proportion of body fat fluctuates with age and that this shift is influenced by gender, race, and individual characteristics at different rates (Borga et al. 2018). Additionally, compared to the conventional BMI method, the BC method better captures the distribution of body fat. A prospective study has shown that higher fat mass is associated with poor CRC prognosis, and BC analysis might be useful in predicting cancer prognosis (He et al. 2021). However, there are still prospects for research on the relationship between BC and CRC risk and its causality. Therefore, using anthropometry, laboratory test findings, retrospective epidemiological analysis, and nutritional risk assessment, we intended to look at the relationship between pertinent BC indicators and CRC.

## Materials and methods

### Study population

This study was a hospital-based case-control study. We selected 303 CRC patients (TNM stage: I=201, II=87, III=15; metastatic disease = 0) who were first diagnosed and hospitalized in the Department of Oncology Surgery, Gastrointestinal Surgery, and Anorectal Surgery of the First Hospital of China Medical University from June 2018 to January 2019 as the case group. In the same period, 286 healthy people from the rehabilitation health care department and physical examination center matched by age and sex to the case group were selected as the control group. All cases were collected in the inpatient department. Controls were healthy people from rehabilitation health care department and physical examination center of the same medical institution, and both cases and controls were local people in urban or rural areas.

Patients were included in case group only if they met all of the following requirements: (a) Patients diagnosed with CRC by pathological biopsy; (b) Age ≥ 18 years old; (c) Patients who were able to complete both the BC measurement and the questionnaire. Patients were excluded from case group if they had any of the following: (a) Non-CRC patients; (b) Suffer from autoimmune diseases; (c) Those who have received anti-tumor treatment; (d) Patients with other tumors or organ dysfunction. Individuals meeting all of the following requirements were included in the control group: (a) Healthy people with no history of malignant tumors; (b) Age ≥ 18 years old; (c) Can complete the BC measurement and questionnaire at the same time. Individuals meeting any of the following exclusion criteria would be excluded from the control group: (a) Suffer from malignant tumors or have a history of malignant tumors; (b) Suffer from autoimmune diseases; (c) Patients with gastrointestinal diseases. Consent for this study was obtained from the ethics committee and all participants signed informed consent forms.

### Data collection for baseline characteristics of the participants and BC measurement

We used a face-to-face questionnaire to obtain the basic information of the participants, including name, sex, age, ethnicity, marital status, education level, living region, first-degree family history of CRC, smoking status, alcohol consumption status, regular physical activity status, sleep time (hours/per day), psychological pressure status, sun exposure status and daily water intake (mL). BC was measured at the same time as the questionnaire survey. We measured BC indicators (BFP, FFMI, and VAI) by bioelectrical impedance analysis, a widely used, non-invasive, simple, and inexpensive method commonly seen in clinical practice (Martin et al. 2013). The instrument we used was the body composition analyzer HBF-701 (Omron Health Medical (China) Co., LTD). FFMI was calculated as (weight (kg) - weight (kg) ×BFP) ÷ height (m)^2^.

### Clinical data collection and nutritional risk assessment of CRC patients

Biochemical indexes and pathological data of CRC patients were obtained through the medical record inquiry system. Biochemical indicators mainly include serum albumin, serum prealbumin, C-reactive protein, and tumor markers. These included carcino-embryonic antigen, carbohydrate antigen-125, carbohydrate antigen-153, carbohydrate antigen-199, etc. All the above were obtained from the first examination after admission without intervention treatment. The nutritional screening tools used in this survey were the ESPEN-recommended Nutritional Risk Screening 2002 and Patient-Generated Subjective Global Assessment, and both tools provide numerical scores, with higher scores indicating poorer nutritional status (De Groot et al. 2020; Lee et al. 2016).

### Statistical analysis

We used Epidata 3.1 software to collate the data and establish the database. SPSS22.0 software was used for statistical analysis. χ^2^ Tests or ANOVA were conducted to compare the baseline characteristics of case and control groups (χ^2^ Tests for categorical variables, ANOVA for continuous variables). The classification of BFP, FFMI, and VAI was as follows: BFP: Low, 5.0%∼9.9% (males)/5.0%∼19.9% (females); Normal, 10.0%∼19.9% (males)/20.0%∼29.9% (females); High, 20.0%∼24.9% (males)/30.0%∼34.9% (females); Higher, ≥ 25% (males)/ ≥35% (females). FFMI: Low, < 17 (males)/ <15 (females)); Normal, ≥ 17 (males)/ ≥15 (females); VAI: Normal, < 10; High, 10 ∼ 15; Higher, ≥ 15. χ^2^ Tests were used to compare the distribution of BFP, FFMI, and VAI in CRC patients with different TNM stages. ANOVA and χ^2^ Tests were used to describe the differences in BFP, FFMI, and VAI between case and control groups for continuous and categorical variables, respectively.

Logistic regression was used to analyze the influence of BFP, FFMI, and VAI on the occurrence of CRC. When BFP was a continuous variable, it was divided into two continuous variables for analysis: normal and low group, high and higher group. The following two adjustment models were used for analysis: Model 1: Adjusted for age and sex. Model 2: Adjusted for age, sex, ethnicity, marital status, education level, living region, smoking status, alcohol consumption status, first-degree family history of CRC, sleep time (hours/per day), sun exposure status, daily water intake, and psychological pressure status. Considering differences in body size, such as differences in body fat distribution (people with normal BFP may have a high visceral fat index, or people with low BFP may have a high visceral fat index). We used logistic regression analysis (univariate analysis and age and sex-adjusted analysis) to further analyze the relationship between BC indicators and CRC risk by combining BFP, FFMI, and VAI in pairwise and overall combinations respectively. Results were expressed as ORs and 95% confidence intervals (95% CIs), with confidence interval values not containing 1 indicating statistical significance. Pearson correlation analysis was used to analyze the correlation between BFP, FFMI, and VAI with biochemical indicators (C-reactive protein, prealbumin, serum albumin, alpha fetal protein, carcino-embryonic antigen, carbohydrate antigen-125, carbohydrate antigen-153, carbohydrate antigen-199) and nutritional risk scores (Nutritional Risk Screening 2002 and Patient-Generated Subjective Global Assessment) in CRC groups. All statistical tests were two-sided, and *P* ≤ 0.05 was considered statistically significant.

## Results

### General characteristics of the study participants

The characteristics of 286 controls (164 males and 122 females) and 303 cases (183 males and 120 females) were displayed in Table 1. The average age of the cases and controls was 62.48 ± 9.04 years and 62.06 ± 8.09 years, respectively. Compared to the control group, the CRC group included more individuals who were over 60 (*P* = 0.019), more members of national minorities (*P* = 0.022), more unmarried people (*P* < 0.001), a lower education level (*P* = 0.050), more urban people (*P* < 0.001), more current-smokers (*P* = 0.034), fewer non-smokers or ex-smokers, more people with a first-degree family history of CRC (*P* = 0.026), shorter sleep duration *P* < 0.001), fewer people with sun exposure (*P* < 0.001), less daily water intake (*P* < 0.001), and greater psychological pressure (*P* < 0.001).

**Table 1:** Characteristics of the participants

Characteristics	Cases (*N*, 303)	Controls (*N*, 286)	*P*-value
Age (years) (N, %)			
Mean ± SD	62.48 ± 9.04	62.06 ± 8.09	0.554
< 60	97 (32.0)	116 (40.6)	**0.019**
≥ 60	206 (68.0)	170 (59.4)	
Sex (N, %)			
Male	183 (60.4)	164 (57.3)	0.252
Female	120 (39.6)	122 (42.7)	
Ethnicity (N, %)			
The Han ethnicity	291 (96.0)	283 (99.0)	**0.022**
Ethnic minority	12 (4.0)	3 (1.0)	
Marital status (N, %)			
Unmarried	51 (6.8)	6 (2.1)	**< 0.001**
Married	252 (93.2)	280 (97.9)	
Education level (N, %)			
Primary school or below	40 (13.2)	24 (8.4)	**0.050**
Middle school	125 (41.3)	111 (38.8)	
High school/secondary school	77 (25.4)	99 (34.6)	
College or more	61 (20.1)	52 (18.2)	
Living region (N, %)			
Rural area	96 (31.7)	150 (52.4)	**< 0.001**
Urban area	207 (68.3)	136 (47.6)	
Smoking status (N, %)			
None	152 (50.2)	155 (54.2)	**0.034**
Ex-smoker	48 (15.8)	60 (21)	
Current-smoker	103 (34.0)	71 (24.8)	
Alcohol consumption status (N, %)			
None	165 (54.5)	166 (58.0)	0.057
Ex-drinker	33 (10.9)	44 (15.4)	
Current-drinker	105 (34.6)	76 (26.6)	
First-degree family history of CRC (N, %)			
Yes	10 (3.3)	2 (0.7)	**0.026**
No	293 (96.7)	284 (99.3)	
Regular physical activity status (N, %)			
Yes	146 (48.2)	135 (47.2)	0.438
No	157 (51.8)	151 (52.8)	
Sleep time (hours/per day)			
Mean ± SD	5.54 ± 1.43	6.82 ± 1.02	**< 0.001**
Sun exposure status (N, %)			
Yes	206 (68)	240 (83.9)	**< 0.001**
No	97 (32)	46 (16.1)	
Daily water intake (N, %)			
≥ 2000 ml	56 (18.5)	8 (2.8)	**< 0.001**
1000 ml ∼ 2000 ml	129 (42.6)	86 (30.1)	
500 ml ∼ 1000 ml	95 (31.4)	140 (49.0)	
< 500 ml	23 (7.5)	52 (18.1)	
Psychological pressure status (N, %)			
Yes	236 (77.8)	155 (54.2)	**< 0.001**
No	67 (22.2)	131 (45.8)	

SD, standard deviation

### Body composition indicators levels in study participants

Table 2 displayed the levels of body composition indicators in patients with CRC and controls. CRC patients had significantly lower levels of BMI and BFP than controls (*P* < 0.001). The proportion of low BFP was higher in the case group than in the control group. However, FFMI and VAI levels were not significantly different between cases and controls.

**Table 2:** Comparison of body composition indicators between case and control group

Characteristics	Cases (*N*, 303)	Controls (*N*, 286)	*P*-value
BFP (N, %)			
Mean ± SD	26.13 ± 5.79	28.69 ± 5.68	**< 0.001**
Low	35 (11.6)	14 (4.9)	**0.001**
Normal	167 (55.1)	154 (53.8)	
High	79 (26.1)	75 (26.2)	
Higher	22 (7.2)	43 (15.1)	
FFMI (N, %)			
Mean ± SD	16.97 ± 2.15	18.66 ± 2.27	0.191
Low	156 (51.5)	133 (46.5)	0.227
Normal	147 (48.5)	153 (53.5)	
VAI (N, %)			
Mean ± SD	8.68 ± 3.9	9.16 ± 3.81	0.129
Normal	119 (39.3)	88 (30.8)	0.094
High	164 (54.1)	175 (61.2)	
Higher	20 (6.6)	23 (8)	
BMI (kg/m^2^), Mean ± SD	23.07 ± 3.21	24.72 ± 3.50	**< 0.001**
Height (cm), Mean ± SD	166.32 ± 7.70	165.95 ± 7.20	0.548

BFP, body fat percentage (Low, 5.0%∼9.9% (males)/5.0%∼19.9% (females); Normal, 10.0%∼19.9% (males)/20.0%∼29.9% (females); High, 20.0%∼24.9% (males)/30.0%∼34.9% (females); Higher, ≥ 25% (males)/ ≥35% (females)); FFMI, fat free mass index (Low, < 17 (males)/ <15 (females); Normal, ≥ 17 (males)/ ≥15 (females)); VAI, visceral adiposity index (Normal, < 10; High, 10 ∼ 15; Higher, ≥ 15); SD, standard deviation; BMI, body mass index

Additionally, the supplementary Table S1 showed the distribution of BFP, FFMI, and VAI in CRC patients with different TNM stages. There was no significant difference in the distribution of BFP, FFMI, and VAI among CRC patients with different stages. The result indicated that BFP, FFMI, and VAI did not correlate with the TNM stage. In other word, the different cancer stages might not be a source of bias in this study.

### Associations between BFP, FFMI, and VAI levels and CRC risk

Table 3 showed ORs and 95% CIs for the association between BFP, FFMI, and VAI and the occurrence of CRC. When BFP, FFMI, and VAI were used as categorical variables, compared with the normal BFP group, the low BFP group showed a higher risk of developing CRC (Model 2: OR = 2.159, 95% CI = 0.937–4.972). High VAI groups showed a higher risk of developing CRC compared with the normal VAI group (Model 2: OR = 1.853, 95% CI = 1.097–3.131). When BFP, FFMI, and VAI were used as continuous variables, the higher the BFP, the lower the risk of CRC (Model 2 (overall): 0.906, 95% CI = 0.860–0.955; Model 2 (normal and low group): OR = 0.869, 95% CI = 0.802–0.943). For each 1 unit increase in FFMI, the risk of CRC decreased by 9.07% (Model 1: OR = 0.903, 95% CI = 0.828–0.984).

**Table 3:** Odds ratios and 95% confidence intervals for the association between BFP, FFMI, and VAI and the risk of CRC

	Categorical ORs (95% CI)		Continuous ORs (95% CI)	
	Model 1	Model 2	Model 1	Model 2
BFP			**Overall: 0.894 (0.857, 0.932)**	**Overall: 0.906 (0.860, 0.955)**
Low	**2.425 (1.252, 4.697)**	2.159 (0.937, 4.972)	**0.856 (0.804, 0.912)**	**0.869 (0.802, 0.943)**
Normal	1 (ref.)	1 (ref.)		
High	0.741 (0.444, 1.236)	0.666 (0.33, 1.344)	0.988 (0.876, 1.113)	0.877 (0.718, 1.070)
Higher	**0.336 (0.168, 0.673)**	0.454 (0.181, 1.14)		
FFMI				
Low	1.317 (0.926, 1.872	1.226 (0.76, 1.977)	**0.903 (0.828, 0.984)**	0.932 (0.832, 1.043)
Normal	1 (ref.)	1 (ref.)		
VAI				
Normal	1 (ref.)	1 (ref.)	0.959 (0.914, 1.007)	0.958 (0.901, 1.019)
High	1.461 (0.992, 2.152)	**1.853 (1.097, 3.131)**		
Higher	0.934 (0.481, 1.816)	0.612 (0.253, 1.483)		

CRC, colorectal cancer; ORs, odd ratios; 95% CI, 95% confidence interval; BFP, body fat percentage (Low, 5.0%∼9.9% (males)/5.0%∼19.9% (females); Normal, 10.0%∼19.9% (males)/20.0%∼29.9% (females); High, 20.0%∼24.9% (males)/30.0%∼34.9% (females); Higher, ≥ 25% (males)/ ≥35% (females)); FFMI, fat free mass index (Low, < 17 (males)/ <15 (females); Normal, ≥ 17 (males)/ ≥15 (females)); VAI, visceral adiposity index (Normal, < 10; High, 10 ∼ 15; Higher, ≥ 15); Model 1: Adjusted for age and sex; Model 2: Adjusted for age, sex, ethnicity, marital status, education level, living region, smoking status, alcohol consumption status, first-degree family history of CRC, sleep time, sun exposure status, daily water intake and psychological pressure status

Table 4 showed ORs and 95% CIs for the association between the pairwise and overall combination of BFP, FFMI, and VAI and the occurrence of CRC. Compared with the Normal BFP + Normal FFMI group, the Low BFP + Normal FFMI group showed a higher risk of developing CRC (Model 1: OR = 2.603, 95% CI = 1.09–6.214), and the High/Higher BFP + Normal FFMI group showed a lower risk of developing CRC (Model 2: OR = 0.384, 95% CI = 0.155–0.951). Compared with the Normal BFP + Normal VAI group, the Normal BFP + High/Higher VAI group showed a higher risk of developing CRC (Model 2: OR = 3.696, 95% CI = 1.856–7.358), and the Low BFP + Normal VAI group showed a higher risk of developing CRC (Model 2: OR = 4.097, 95% CI = 1.56-10.761). Compared with the Normal VAI + Normal FFMI group, the High/Higher VAI + Low FFMI group showed a higher risk of developing CRC (Model 2: OR = 3.144, 95% CI = 1.138–8.686). Compared with the Normal BFP + Normal FFMI + Normal VAI group, the Normal BFP + Normal FFMI + High/Higher VAI group showed a higher risk of developing CRC (Model 2: OR = 4.849, 95% CI = 1.427–16.474), and the Low BFP + Normal FFMI + Normal VAI group showed a higher risk of developing CRC (Model 2: OR = 6.452, 95% CI = 1.216–34.228).

**Table 4:** Odds ratios and 95% confidence intervals for the association between BFP, FFMI, and VAI (three indicators combine in pairs and overall combinations) and the occurrence of CRC

	*N*, cases/controls	ORs (95% CI)		
		Univariate analysis	Model 1	Model 2
BFP + FFMI	303/286			
A0 + B0	15/6	2.554 (0.95, 6.866)	2.475 (0.918, 6.668)	2.049 (0.604, 6.954)
A0 + B1	20/8	**2.554 (1.072, 6.085)**	**2.603 (1.09, 6.214)**	1.930 (0.652, 5.713)
A1 + B0	74/59	1.281 (0.82, 2.001)	1.188 (0.736, 1.916)	0.827 (0.435, 1.573)
A1 + B1	93/95	1 (ref.)	1 (ref.)	1 (ref.)
A2/A3 + B0	67/68	1.006 (0.647, 1.566)	0.814 (0.424, 1.564)	0.629 (0.253, 1.566)
A2/A3 + B1	34/50	0.695 (0.413, 1.17)	0.587 (0.302, 1.142)	**0.384 (0.155, 0.951)**
BFP + VAI	303/286			
A0 + C0	31/14	**3.389(1.639, 7.009)**	**4.425(2.061, 9.501)**	**4.097 (1.56, 10.761)**
A0 + C1/C2	4/0	-	-	
A1 + C0	49/75	1 (ref.)	1 (ref.)	1 (ref.)
A1 + C1/C2	118/79	**2.286 (1.445, 3.618)**	**3.168 (1.886, 5.323)**	**3.696 (1.856, 7.358)**
A2/A3 + C0	84/86	1.495 (0.935, 2.39)	0.932 (0.531, 1.638)	0.935 (0.427, 2.051)
A2/A3 + C1/C2	17/32	0.813 (0.408, 1.621)	0.599 (0.289, 1.242)	0.644 (0.257, 1.61)
VAI + FFMI	303/286			
C0 + B0	120/120	1.250 (0.781, 2.001)	1.196 (0.734, 1.949)	1.502 (0.779, 2.894)
C0 + B1	44/55	1 (ref.)	1 (ref.)	1 (ref.)
C1/C2 + B0	36/13	**3.462 (1.639, 7.313)**	**3.431 (1.607, 7.325)**	**3.144 (1.138, 8.686)**
C1/C2 + B1	103/98	1.314 (0.81, 2.13)	1.303 (0.795, 2.136)	1.811 (0.934, 3.511)
BFP + FFMI + VAI	158/164			
A0 + B1 + C0	17/8	**6.109 (1.909, 19.554)**	**6.293 (1.962, 20.186)**	**6.452 (1.216, 34.228)**
A1 + B0 + C0	41/52	2.267 (0.919, 5.59)	1.55 (0.582, 4.128)	1.778 (0.466, 6.781)
A1 + B1 + C0	8/23	1 (ref.)	1 (ref.)	1 (ref.)
A1 + B1 + C1/C2	73/57	**3.682 (1.533, 8.841)**	**3.734 (1.551, 8.990)**	**4.849 (1.427, 16.474)**
A2/A3 + B1 + C0	19/24	2.276 (0.833, 6.216)	1.163 (0.351, 3.852)	1.612 (0.263, 9.894)

CRC, colorectal cancer; ORs, odd ratios; 95% CI, 95% confidence interval; BFP, body fat percentage (A0, Low, 5.0%∼9.9% (males)/5.0%∼19.9% (females); A1, Normal, 10.0%∼19.9% (males)/20.0%∼29.9% (females); A2, High, 20.0%∼24.9% (males)/30.0%∼34.9% (females); A3, Higher, ≥ 25% (males)/ ≥35% (females)); FFMI, fat free mass index (B0, Low, < 17 (males)/ <15 (females); B1, Normal, ≥ 17 (males)/ ≥15 (females)); VAI, visceral adiposity index (C0, Normal, < 10; C1, High, 10 ∼ 15; C2, Higher, ≥ 15); Model 1: Adjusted for age and sex; Model 2: Adjusted for age, sex, ethnicity, marital status, education level, living region, smoking status, alcohol consumption status, first-degree family history of CRC, sleep time, sun exposure status, daily water intake and psychological pressure status

### Correlation of BFP, FFMI, and VAI levels with biochemical indicators and nutritional risk scores in CRC patients

Table 5 showed the correlation of BFP, FFMI, and VAI with biochemical indicators (C-reactive protein, prealbumin, serum albumin, alpha fetal protein, carcino-embryonic antigen, carbohydrate antigen-125, carbohydrate antigen-153, carbohydrate antigen-199), Nutritional Risk Screening 2002, and Patient-Generated Subjective Global Assessment of CRC patients. For overall patients, FFMI was positively correlated with VAI and negatively correlated with BFP. FFMI and VAI were positively correlated with prealbumin and serum albumin, and negatively correlated with Nutritional Risk Screening 2002 and Patient-Generated Subjective Global Assessment. There was no correlation between BFP and biochemical indicators or nutritional risk scores in CRC patients. For male patients, BFP was positively correlated with VAI and prealbumin, and negatively correlated with BFP, Nutritional Risk Screening 2002, and Patient-Generated Subjective Global Assessment. FFMI was positively correlated with VAI, prealbumin, and serum albumin and negatively correlated with Nutritional Risk Screening 2002 and Patient-Generated Subjective Global Assessment. VAI was positively correlated with serum albumin and negatively correlated with carbohydrate antigen-199, Nutritional Risk Screening 2002, and Patient-Generated Subjective Global Assessment. For female patients, BFP was positively correlated with VAI. VAI was positively correlated with BFP and FFMI, prealbumin, and serum albumin and negatively correlated with Nutritional Risk Screening 2002 and Patient-Generated Subjective Global Assessment. FFMI was negatively correlated with Nutritional Risk Screening 2002 and Patient-Generated Subjective Global Assessment. VAI was negatively correlated with Nutritional Risk Screening 2002.

**Table 5:** Correlation of BFP, FFMI, and VAI with biochemical indicators and nutritional risk scores in CRC patients

		BFP	FFMI	VAI	CRP	PA	ALB	AFP	CEA	CA-125	CA-153	CA-199	NRS2002	PG-SGA
Overall	BFP	1.000	**-0.182** ^**^	-0.002	-0.083	0.040	0.045	-0.044	-0.074	-0.016	0.000	0.065	-0.006	-0.037
	FFMI		1.000	**0.724** ^**^	0.053	**0.154** ^**^	**0.136** ^*^	-0.038	0.024	0.001	-0.034	-0.033	**-0.358** ^**^	**-0.300** ^**^
	VAI			1.000	0.025	**0.145** ^*^	**0.121** ^*^	0.030	-0.012	-0.023	-0.032	-0.011	**-0.303** ^**^	**-0.240** ^**^
Males	BFP	1.000	**-0.210** ^**^	**0.610** ^**^	-0.018	**0.161** ^*^	0.133	0.005	-0.088	-0.076	-0.081	-0.125	**-0.196** ^**^	**-0.170** ^*^
	FFMI		1.000	**0.636** ^**^	0.048	**0.153** ^*^	**0.154** ^*^	-0.099	-0.002	0.012	-0.030	-0.052	**-0.409** ^**^	**-0.352** ^**^
	VAI			1.000	0.005	0.130	**0.177** ^*^	0.001	-0.045	-0.022	-0.045	**-0.180** ^*^	**-0.318** ^**^	**-0.318** ^**^
Females	BFP	1.000	0.152	**0.720** ^**^	-0.082	0.091	0.006	-0.090	0.045	-0.042	0.167	0.132	-0.036	0.042
	FFMI		1.000	**0.539** ^**^	-0.051	0.103	0.134	0.132	0.050	0.027	-0.029	0.017	**-0.236** ^**^	**-0.264** ^**^
	VAI			1.000	-0.085	0.109	0.064	0.022	-0.016	0.021	0.179	0.109	**-0.209** ^*^	-0.135

CRC, colorectal cancer; BFP, body fat percentage; FFMI, fat free mass index; VAI, visceral adiposity index; CRP, C-reactive protein; PA, prealbumin; ALB, albumin; AFP, alpha fetal protein; CEA, carcino-embryonic antigen; CA-125, carbohydrate antigen-125; CA-153, carbohydrate antigen-153; CA-199, carbohydrate antigen-199; NRS2002, Nutritional Risk Screening 2002; PG-SGA, Patient-Generated Subjective Global Assessment. *, *P* < 0.05; **, *P* < 0.01

## Discussion

Our findings suggested a considerable correlation between different classifications of BC indices (low BFP and high VAI) and a higher risk of CRC. Furthermore, there were favorable connections between FFMI and VAI and the nutritional risk scores (Nutritional Risk Screening 2002 and Patient-Generated Subjective Global Assessment) and biochemical indicators (prealbumin and serum albumin) of CRC patients.

BFP refers to the weight of body fat as a proportion of body weight, which represents the degree of total body fat accumulation. The low total body fat might be due to malnutritioncaused by insufficient carbohydrate and fat intake and cancer cachexia. Studies have pointed out that the great majority of cancer patients have a certain degree of malnutrition (Bossi et al. 2021) and cancer cachexia (Vudatha et al. 2022; Tichy and Parry 2023). CRC has a wasting nature, and there may be a potential wasting process that could cause malnutrition and cancer cachexia before the cancer diagnosis. Cachexia, a disease-related malnutrition, is considered a state of systemic failure as a late consequence of diseases such as cancer, organ failure, or infection, which can lead to significant morbidity and mortality (Meza-Valderrama et al. 2021; Ferrer et al. 2023). Cachexia is also a complex metabolic syndrome characterized by a decrease in skeletal muscle mass with or without a decrease in fat mass. CRC is expendable, and some patients are at an advanced stage when they are first diagnosed, such patients are often prone to cachexia (Nishikawa et al. 2021), resulting in a lower BFP. The reason for the association of low BFP and higher CRC risk in this case-control study may be tumor cachexia. Thus, in the future, body composition measurements could be used to monitor the physical status of patients with cancer cachexia as well as healthy people.

VAI represents the degree of visceral fat accumulation to a certain extent, which can increase the burden of visceral organs and cause abdominal organ dysfunction (Amato et al. 2010). Studies have suggested that visceral obesity may be more carcinogenic than total body fat and is associated with cancer and metabolic diseases such as breast cancer, type 2 diabetes mellitus, metabolic syndrome, hypertension, etc. (Amato et al. 2010; Du et al. 2014; Godinho-Mota et al. 2018). In this study, whether VAI was analyzed alone or combined with BFP and FFMI, it was concluded that high VAI could increase the risk of CRC. This result can be supported by the following points. First, it has been reported that fat accumulation is inversely related to adiponectin. Adiponectin can inhibit the occurrence of CRC through the AMPK/mTOR pathway (Macleod et al. 2023; Otake et al. 2005). The higher the VAI, the lower the adiponectin, and thus the more likely it is to lead to the occurrence of CRC. Second, studies have reported a close link between visceral fat and oxidative stress, which can promote cancer by causing DNA damage (Matsuzawa-Nagata et al. 2008; Van der et al. 2005). Also, the chronic inflammatory state within adipose tissue can lead to oxidative stress, which is a potential factor for carcinogenesis and tumor cell proliferation (Reuter et al. 2010). Third, oxidative stress is also closely related to insulin resistance. Insulin resistance induces hyperinsulinemia and increases the level of insulin-like growth factor, which can activate the Akt/S6K/PI3K/mTOR signaling pathway in cancer (Huang et al. 2019). Therefore, visceral fat may cause the occurrence of CRC by promoting oxidative stress. Lastly, adipocytokines are related to the occurrence of CRC (Riondino et al. 2014). The association between adipose tissue and cancer may be explained by the metabolic anomalies in adipose tissue that impact the release of several hormones, adipokines, inflammatory agents, enzymes, free fatty acids, and growth factors (Kim and Scherer 2021). Additionally, several research has shown that VAI has a positive correlation with TNF-α/C-reactive protein /IL-6 and that elevated visceral fat can stimulate the release of adipocytokines. However, adipokine secretion dysregulation can also lead to insulin resistance and worsen cancerous situations (Riondino et al. 2014). Therefore, visceral fat might cause CRC by promoting the release of adipocytokines. Meanwhile, considering that this study was a case-control study, the results obtained could not guarantee that the increase in visceral fat occurred before the occurrence of CRC. However, the result of this study was surprising, which might be cancer cachexia had little influence on visceral fat. Therefore, visceral fat is a relatively stable indicator that can be used as an index of body composition to predict the occurrence of CRC.

FFMI is a crucial BC indicator that may be used to assess a person’s non-fat weight and has been demonstrated to be a measure of muscle mass (Landi et al. 2019). Prior research has indicated that FFMI is positively correlated with the prognosis of cancer and adversely correlated with survival (Cederholm et al. 2017). Furthermore, a decrease in fat-free body mass accounts for around 70% of the weight loss experienced by cancer patients (Lonbro et al. 2013). Additionally, research has demonstrated that low FFMI is frequently a sign of malnutrition and that loss of muscle mass is a risk factor for CRC (Deutz et al. 2019). When FFMI was employed as a continuous parameter in our study, it showed a negative correlation with the incidence of CRC (Table 3). As we mentioned earlier, loss of muscle mass was a hallmark of cancer cachexia (Nishikawa et al. 2021). Low FFMI in CRC patients might also signal cancer cachexia, which might be present at the time of diagnosis and result in a lower FFMI outcome in the case group. This suggested that FFMI may be a predictor of cancer cachexia. This was consistent with the findings of earlier research on the relationship between FFMI and cancer prognosis (Zhang et al. 2021). Nevertheless, the data indicated that FFMI did not appear to have a substantial impact on the incidence of CRC when paired with BFP and VAI. The tiny sample size under the Normal BFP + Low FFMI + Normal VAI categorization may be the cause of this. We think that FFMI affects the incidence and prognosis of CRC, and more research on the connection between FFMI and CRC is still necessary.

Malnutrition is an indicator in the majority of malignant tumors, and digestive tract cancers in particular are more serious. The prognosis for CRC is strongly correlated with malnutrition. Clinical nutrition assessment indicators frequently use prealbumin, serum albumin, Nutritional Risk Screening 2002, and Patient-Generated Subjective Global Assessment (De Groot et al. 2020; Lee et al. 2016). FFMI and VAI were favorably linked with serum prealbumin, serum albumin levels, Nutritional Risk Screening 2002, and Patient-Generated Subjective Global Assessment in our study, which suggested that FFMI and VAI were related to patients’ nutritional status. Qiangsheng He’s study revealed that a higher ratio of fat mass to fat-free mass was associated with cancer prognosis, and BC measurements of fat mass and fat-free mass could provide useful information on clinical health status (He et al. 2021). Therefore, it will also be easier, faster, and safer for patients to assess their nutritional status using FFMI and VAI, which will be helpful for the nursing, rehabilitation, and prognosis monitoring of CRC patients.

There were still some limitations in our study. First, memory bias might been introduced by the retrospective questionnaire survey that we employed to gather data. Secondly, we were unable to investigate the impact of BC indexes on the biochemical indicators and nutritional status of healthy individuals since we were only able to gather pertinent biochemical indexes and nutritional score indexes for the case group. Thirdly, case-control studies might be subject to reverse causation and could not guarantee the order of causation, limiting the effectiveness of cancer prevention. Additional studies like prospective studies are required to examine the effects of BC on human health in the future.

## Conclusion

To sum up, our research revealed a correlation between a high risk of CRC and low BFP and high VAI. VAI has proven to be a reasonably reliable predictor for predicting the occurrence of CRC, highlighting its critical role in directing CRC treatment and prevention initiatives. Furthermore, it was discovered that FFMI and VAI were associated with the nutritional status of CRC patients, providing important insights for tracking their nutritional health. In the context of CRC, these findings have important ramifications for prognostic surveillance, rehabilitation initiatives, and patient care.

### Electronic supplementary material

Below is the link to the electronic supplementary material.

Supplementary Material 1

## Data Availability

No datasets were generated or analysed during the current study.
