# Supplementary material for: Association of body composition indicators with colorectal cancer: a hospital-based case-control study
Source: J Cancer Res Clin Oncol. 2024 Jul 9;150(7):344. doi: 10.1007/s00432-024-05866-4 (PMC11233301; doi:10.1007/s00432-024-05866-4)
Supplement: Supplementary file 1 — Supplementary Material 1 [file 432_2024_5866_MOESM1_ESM.docx]

Supplementary materials

Table S1 Comparison of body composition indicators in patients with different TNM stages

| Characteristics | TNM stage | | | *P-*value |
| --- | --- | --- | --- | --- |
|  | Ⅰ (N, 201) | Ⅱ (N, 87) | Ⅲ (N, 15) |  |
| BFP (N, %) |  |  |  | 0.171 |
| Low | 119 (59.2) | 42 (48.3) | 6 (40.0) |  |
| Normal | 20 (10.0) | 11 (12.6) | 4 (26.7) |  |
| High | 46 (22.9) | 28 (32.2) | 5 (33.3) |  |
| Higher | 16 (8.0) | 6 (6.9) | 0 (0.0) |  |
| FFMI (N, %) |  |  |  | 0.064 |
| Low | 90 (44.8) | 46 (52.9) | 11 (73.3) |  |
| Normal | 111 (55.2) | 41 (47.1) | 4 (26.7) |  |
| VAI (N, %) |  |  |  | 0.251 |
| Normal | 104 (51.7) | 49 (56.3) | 11 (73.3) |  |
| High | 84 (41.8) | 33 (37.9) | 2 (13.3) |  |
| Higher | 13 (6.5) | 5 (5.7) | 2 (13.3) |  |

BFP: body fat percentage; FFMI: fat free mass index; BMI: body mass index; VAI: visceral adiposity index.
